# Systemic Venous Excess in Acute Heart Failure: A Retrograde Signal of Right Ventricular Uncoupling

**DOI:** 10.1016/j.jacadv.2026.103322

**Published:** 2026-09-22

**Authors:** José Carlos Gasca-Aldama, Osvaldo Alexis Marché-Fernández, José Ernesto Juárez León

**Affiliations:** a Intensive Care Unit, Hospital Juárez de México, Mexico City, Mexico; b Cardiopulmonary Physiology Clinic, Hospital Juárez de México, Mexico City, Mexico; c Lung Clinic, Hospital Star Médica Santa Fe, Mexico City, Mexico

We read with interest the study by Araiza-Garaygordobil et al^1^ demonstrating that an admission Venous Excess Ultrasound Score (VExUS) ≥2 is strongly associated with acute kidney injury in acute heart failure. Although the authors conceptualize VExUS as a passive marker of volume-driven congestion, we argue that systemic venous excess represents the retrograde hemodynamic footprint of ventriculoarterial uncoupling.^2^

In acute heart failure with reduced ejection fraction (median left ventricular ejection fraction 24%), the primary driver is postcapillary uncoupling (group 2 pulmonary hypertension). Their data on left ventricular diastolic function—specifically the restrictive pattern shown by a higher mitral E/A ratio in the VExUS ≥2 group (1.63 vs 1.20, *P* = 0.049)^1^—indicate severe elevation in left-sided filling pressures, passively increasing right ventricular (RV) afterload (arterial elastance, Ea).

Concurrently, systolic ventricular interdependence and myocardial impairment depress RV contractility (end-systolic elastance). Although they evaluated RV parameters in isolation, their data allow estimation of RV-PA coupling via the tricuspid annular plane systolic excursion (TAPSE)/pulmonary artery (PA) systolic pressure (PASP) ratio.^3^ Using median tricuspid regurgitation velocity (2.95 vs 2.63 m/s) and estimated right atrial pressure (15 vs 8 mm Hg) to calculate PASP (4∗tricuspid regurgitation velocity 2 + right atrial pressure),^1^ we infer a TAPSE/PASP ratio of 0.33 mm/mm Hg in the VExUS ≥2 group, compared to 0.49 mm/mm Hg in the VExUS 0 to 1 group.

An inferred TAPSE/PASP of 0.33 mm/mm Hg sits below the prognostic threshold (<0.38 mm/mm Hg) validated against invasive pressure-volume loops by Schmeisser et al.^3^ Although derived from cohort-level summary statistics rather than individual measurements and thus serving as an approximation, this value supports the hypothesis of severe ventriculoarterial uncoupling. Under a conceptual physiologic model of venous capacitance, when RV-PA uncoupling occurs, the rising right-sided filling pressures are hypothesized to narrow the pressure gradient for venous return. If effective cardiac output falls below venous return, a positive venous accumulation rate (venous accumulation rate = venous return − cardiac output) ensues,^4^ generating the retrograde venous stasis captured by VExUS.

This uncoupling-driven stasis provides a plausible conceptual link to renal injury, consistent with the 4.6-fold higher risk of acute kidney injury observed in the VExUS ≥2 group,^1^ of whom 53% exhibited a monophasic renal Doppler pattern. Physiologically, this high-stasis state—the spectral equivalent of the highest tertile of the Renal Venous Stasis Index (>0.32) described by Husain-Syed et al^5^—is hypothesized to increase renal interstitial pressure within the encapsulated kidney, opposing the glomerular filtration gradient and predisposing patients to congestion-associated nephropathy.

In conclusion, VExUS is an exceptional bedside tool. However, its true value lies not in measuring volume, but in serving as a backward alarm of forward ventriculoarterial uncoupling.
